# Synthesis and Characterization of Bismuth-Cerium Oxides for the Catalytic Oxidation of Diesel Soot

**DOI:** 10.3390/ma13061369

**Published:** 2020-03-18

**Authors:** Sabrina C. Hebert, Klaus Stöwe

**Affiliations:** Faculty of Natural Science, Professorship of Chemical Technology, Chemnitz University of Technology, 09111 Chemnitz, Germany

**Keywords:** bismuth-cerium-oxides, co-precipitation, reverse strike precipitation, diesel soot oxidation, dynamic oxygen storage capacity

## Abstract

In this paper, the syntheses of a set of cerium-bismuth mixed oxides with the formula Ce_1−x_Bi_x_O_2−x/2_, where the range of x is 0.0 to 1.0 in 10 mol% steps, via co-precipitation methods is described. Two synthesis routes are tested: The “normal” and the so called “reverse strike” (RS) co-precipitation route. The syntheses are performed with an automated synthesis robot. The activity for Diesel soot oxidation is measured by temperature programmed oxidation with an automated, serial thermogravimetric and differential scanning calorimetry system (TGA/DSC). P90 is used as a model soot. An automated and reproducible tight contact between soot and catalyst is used. The synthesized catalysts are characterized in terms of the specific surface area according to Brunauer, Emmett and Teller (*S_BET_*), as well as the dynamic oxygen storage capacity (*OSC_dyn_*). The crystalline phases of the catalysts are analysed by powder X-ray diffraction (PXRD) and Raman spectroscopy. The elemental mass fraction of the synthesized catalysts is verified by X-ray fluorescence (XRF) analysis. A correlation between the *T_50_* values, *OSC_dyn_* and *S_BET_* has been discovered. The best catalytic performance is exhibited by the catalyst with the formula RS-Ce_0.8_Bi_0.2_O_x_ which is synthesized by the reverse strike co-precipitation route. Here, a correlation between activity, *OSC_dyn_*, and *S_BET_* can be confirmed based on structural properties.

## 1. Introduction

Diesel soot has proven to be carcinogenic [1] and furthermore, it can influence the environment, the vegetation, or the climate [2]. Diesel soot may cause lung or cardiovascular diseases [3,4]. Therefore, different technologies to control the emission of Diesel soot have been developed. Modern Diesel vehicles possess a Diesel particulate filter (DPF) system to filtrate Diesel soot from the exhaust stream. Over time, Diesel soot accumulates on the surface or in the channels of the DPF. The accumulated Diesel soot causes an increase in pressure drop, and this influences the fuel consumption or leads to filter failure [2]. In the absence of a filter regeneration process, the car engine will stop due to DPF plugging [5]. Today, active and passive regeneration processes are established. Another possibility is the use of fuel born catalysts based on Ce or Fe [6]. During the active regeneration process, the accumulated soot is periodically combusted in an oxidising atmosphere when the pressure drop reaches a preset limit. The heat for this process is generated by an electric heater, a flame-based burner, or microwave cavity [7]. At the active regeneration process, temperatures above 600 °C are reached. This causes on the one hand problems with materials of the DPF because of their melting point [8]. On the other hand, large amounts of energy are needed [9]. Therefore, the filter wall is coated with a catalyst [2]. During the passive regeneration, the accumulated soot is combusted continuously by chemical reaction. Here, no additional amount of fuel is needed and the oxidation temperature is equal to the exhaust temperature. The problem of the passive regeneration is the suitability and costs of catalysts. Common catalysts are based on precious metals [2].

Many researchers investigate soot oxidation with ceria as catalyst applied in three-way catalytic converters, in solid oxide fuel cells fed with hydrocarbons, or in the water-gas-shift reaction [10]. Ceria has the ability to incorporate oxygen vacancies into the lattice resulting in a substoichiometric phase CeO_2−x_ through a reduction process [11]. The Ce_1−x_Bi_x_O_2−x/2_-system is a promising candidate for the passive regeneration of the DPF [12]. This mixed oxide system is free of precious metals. The investigation of this system is directly related to the introduction of vacancies in oxide ion sublattice. Oxide ion vacancies are inherent to ceria. Additionally, doping with ions with a lower formal charge than in Ce^4+^ and an asymmetric coordination sphere like Bi^3+^ ions increases the amount of oxide ion vacancies and reducibility of the oxide. Via s-p-hybridisation in the formal electron configuration of Bi^3+^ [Xe] 4f^14^5d^10^6s^2^6p^0^ the coordination sphere of this ion is typically distorted (so-called lone pair) [10]. Dikmen et al. observed a high oxide ion conductivity for Bi-doped CeO_2_. They synthesized Bi-Ce mixed oxides via co-precipitation and subsequent hydrothermal aging at *T* = 900 – 1300 °C [13]. *Zhao* and *Feng* doped CeO_2_ with Bi^3+^ and M^2+^ (M = Ca^2+^, Sr^2+^, Ba^2+^). All of their synthesized oxides possess a high oxide ion conductivity. The highest oxide ion conductivity showed the following oxide: Ce_0.95_Ca_0.05_Bi_0.4_O_2.55_ [14]. Hund et al. synthesized Bi^3+^-doped CeO_2_ via solid state reaction at *T* = 800 °C. They found up to 40 mol% Bi^3+^ exclusively as fluorite-type CeO_2_ crystalline phase but with increased lattice constant of the cubic mixed oxide phase because of Bi^3+^ insertion [15]. A more detailed investigation of the crystal structure of Bi^3+^-doped CeO_2_ has not yet been made. This is because all the prepared Bi-Ce mixed oxides have nanocrystalline structures [10] and these reveal very broad reflections in PXRD pattern. First attempts to clarify the structure are made with the combination of extended X-ray absorption fine structure (EXAFS) and PXRD by Frolova et al. They discovered that the introduction of bismuth oxide in CeO_2_ leads to the formation of a single-phase solid solution system. The resulting structure is close to the fluorite-type structure of CeO_2_. With increasing Bi content, the unit cell parameter and the disorder of the structure increases [16]. Sardar et al. synthesized mixed oxides via hydrothermal synthesis starting from cerium chloride and sodium bismutate at *T* = 240 °C. They investigated the structure through XRD and X-ray absorption near-edge fine structure (XANES) measurements. They conclude that the mixed oxides have the formula ${\mathrm{Ce}}_{1-x}^{4+}{\mathrm{Bi}}_{x}^{3+}O_{2-0,5x}$ (x ≤ 0.6) and reveal a locally distorted fluorite-type structure because of the unsymmetrical coordination of Bi^3+^ [10].

In this paper, a series of Bi-Ce mixed oxides with varying molar ratio between Ce and Bi are synthesized automatically via the synthesis robot Chemspeed Accelerator SLT 106. Two routes of co-precipitation are used: The normal co-precipitation route and the reverse strike co-precipitation route according to *Lee at al.* [17]. In the normal co-precipitation route, the precipitation agent is added to the metal salt precursor solution. The reaction runs from low to high pH values. Due to hydrolysis reactions, we have to deal with different beginnings of precipitation for different precursors. For the reverse strike co-precipitation route, the premixed precursor solution is added to the precipitation agent. Thus, the reaction starts in the basic pH range and ends at pH = 6. This method guarantees a homogenous start of the precipitation and prevents the oligomerisations of hydrated bismuth oxides in the acidic pH range [17,18]. The catalysts are characterized via PXRD, Raman spectroscopy, X-ray fluorescence (XRF), and specific surface measurements according to Brunauer, Emmett, and Teller (*S_BET_*). The catalytic activity for Diesel soot oxidation and dynamic oxygen storage capacity *(OSC_dyn_)* are measured by automated serial thermogravimetric analysis methods.

## 2. Materials and Methods 

### 2.1. Catalyst Preparation

The chemicals specified below were used without any further purification.

A set of cerium-bismuth mixed oxides with the formula Ce_1−x_Bi_x_O_2−x/2_ in the range of 0.0 ≤ x ≤ 1.0 in 10 mol% steps were prepared via co-precipitation methods. The syntheses were performed with the synthesis robot Accelerator SLT106 from Chemspeed Technologies AG, Switzerland. Two co-precipitation synthesis routes were used: The normal co-precipitation with continuous addition of the precipitation agent via a 4-needle head (4NH) of the synthesis robot for liquid dosing to a given solution mixture of both metal salts and the reverse strike co-precipitation route according to Lee et al. [17] with reversal of this sequence of solution combination. The syntheses were performed in lined beakers with magnetic stirring bars, which were tempered via a thermostat from Huber Corp. to a temperature of *T* = 25 °C. The control of the thermostat was implemented in the software “ApplicationExecutor” for the synthesis robot. During the normal co-precipitation route, 0.5 M Ce(NO_3_)_3_·6H_2_O and 0.5 M Bi(NO_3_)_3_·5H_2_O (99.5%, Alfa Aesar) were dissolved in 1 M HNO_3_ and transferred in beakers as reservoirs. Via the 4NH of the synthesis robot, the corresponding molar amounts of the solutions were pipetted into lined beakers, mixed by magnetic stirring and kept at *T* = 25 °C. In a different lined beaker the precipitant, in our case a 1 M (NH_4_)_2_CO_3_ (food grade, BASF) solution, which was dissolved in ultra-pure water, was also brought to a temperature *T* = 25 °C. Via 4NH, the precipitant was transferred to the premixed nitrate solutions. The white to pale yellow precipitations were stirred for a further *t* = 0.5 h at *T* = 25 °C. After stirring, the precipitations were separated from the supernatants via a 3-fold parallel pressure filtration station at an overpressure of *p* = 1 bar. Polyethersulfone membranes (Sartorius Stedim Biotech Corp.) with 0.1 µm mean pore size were used for filtration. The filtrated samples were dried over night at room temperature, crushed and ground in an agate mortar until fine powders were received and subsequently calcined at *T* = 800 °C for *t* = 5 h. During the reverse strike co-precipitation route the corresponding molar amounts of the nitrates were premixed. The premixed nitrate solution was continuously dosed via the 4NH to the tempered precipitant until a preset pH was achieved (pH = 6). The subsequent downstream processes of stirring, filtration, and calcination were equal to the normal co-precipitation route. The dispensing and aspirating speeds are summarized in Table 1.

### 2.2. Activity Measurements—Soot Oxidation and OSC_dyn_

The catalytic performance for soot oxidation of the synthesized catalysts was investigated by automated serial thermogravimetric analyses coupled with heat flow differential scanning calorimetry (TGA-DSC 1 1600; Mettler Toledo Corp.). The model soot used was the carbon black P90 supplied by Evonik Degussa. In this work, the so-called tight contact in an automated mode was used. The tight contact was realized with an asymmetric dual centrifuge in a highly reproducible mode of operation. For this purpose, the Speedmixer^TM^ from Hauschild Corp. was used. The soot and catalyst with a weight ratio of 1:4 were mixed for *t* = 300 s at a rotation speed of *rs* = 3000 rpm. Ca. *m* = 10 mg of the mixed soot and catalyst was placed in a corundum crucible of volume *V* = 70 µl and heated to *T* = 700 °C at a heating rate of *r* = 5 °C·min^−1^ with a synthetic air flow of $\dot{V}$ = 25 mL·min^−1^. The activity of the catalyst was determined by the characteristic combustion temperature at a specific weight loss. In this work, the *T_50_* temperatures were used. These were the temperatures where 50% of the soot was oxidized. These values were determined with the help of software Star^e^ from Mettler Toledo Corp.

Another significant property for soot oxidation catalysts is the dynamic oxygen storage capacity (*OSC_dyn_*). In this work, *OSC_dyn_* was determined with the help of the TGA-DSC11600 from Mettler Toledo Corp., described above. To calculate the *OSC_dyn_*, two heating and cooling cycles were performed. First, m = 30 mg of the catalyst which was calcined at T = 800 °C was placed in a *V* = 70 µl corundum crucible and heated to T = 700 °C under a nitrogen flow of $\dot{V}$ = 25 mL·min^−1^ to release adsorbed oxygen, water, or other compounds. The system was stabilized for *t* = 10 min. Next, the system was cooled to *T* = 150 °C under a synthetic air flow of $\dot{V}$ = 25 mL·min^−1^ for oxygen uptake and stabilized for *t* = 10 min. This procedure was repeated and the weight loss of the second heating step was used for calculating the *OSC_dyn_* as mass difference of $\frac{m_{O_{2}}^{150\;{}^{\circ}C}{-m}_{N_{2}}^{700\;{}^{\circ}C}}{M_{O_{2}}\cdot m_{cat}}$, where $m_{O_{2}}^{150\;{}^{\circ}C}$ is the mass of catalyst at a temperature *T* = 150 °C after oxygen uptake,$\;m_{N_{2}}^{700\;{}^{\circ}C}$ is the mass of catalyst at *T* = 700 °C after oxygen release,$\;M_{O_{2}}$ is the molar mass of oxygen, and $m_{cat}$ is the buoyancy-corrected mass of catalyst weighed in a *V* = 70 µl corundum crucible after desorption of compounds like water. *OSC_dyn_* is represented in ${µmol}_{O_{2}}\cdot g_{cat}^{-1}$. For measuring the *OSC_dyn_* a heating/cooling rate of *r* = 5 °C·min^−1^ was used.

### 2.3. Catalyst Characterisation

Powder X-Ray diffraction (PXRD) was performed on a Bruker D8 diffractometer with Co fine focus X-ray source (Ni filter, *λ_K__α_* = 1.79021 Å, Θ-Θ geometry, VDS, Lynxeye detector) for samples of the normal synthesis route and on a STOE-Stadi P with Cu fine focus X-ray source (monochromator, *λ_K__α__1_* = 1.54056 Å, Θ-Θ geometry, MYTHEN2 R1K detector) for samples of the reverse strike synthesis route. The selected intensity data were collected in the 2θ range from 20 to 85°. Qualitative phase identification was achieved by diffraction pattern assignment according to ICDD data (International Centre for Diffraction Data). Following ICDD data are used: CeO_2_ ICDD#75-76, α-Bi_2_O_3_ ICDD#71-2274, and β-Bi_2_O_3_ ICDD#78-1793. Crystalline phases were identified through Rietveld refinement with the program TOPAS version 4.2 according to [19,20,21] adapted for the cerium-bismuth mixed oxides. Instrumental parameters for the fundamental parameter TOPAS refinement were determined by refinement of a LaB_6_ reference sample diffraction pattern.

Raman spectra were recorded with an inVia Raman Microscope from Renishaw Corp. A frequency-doubled Nd:Yag laser of the model RL473C from Renishaw with a wavelength of *λ* = 532 nm and a UVDD-CCD-Array detector with a grid consisting of 1800 lines/mm was used. The laser power depends on the measuring time. Usually laser powers of 0.5% of the maximal laser power *P* = 50 mW and a measuring time of *t* = 60 s were employed. The calcined samples were placed on a 96-well microplate with V-shaped bottom from Greiner Corp. The microscope was used in line focus. The sample were measured at three different points.

The determination of the specific surface according to Brunauer, Emmett and Teller was performed on a NOVA touch LX4 surface area and pore size analyzer from Quantachrome Corp. The evaluation was performed with the program TouchWin^TM^ version 1.11 from Quantachrome Corp. The specific surface was measured by N_2_ adsorption-desorption isotherms at the temperature of liquid N_2_ (*T* = −196 °C). The samples were degassed under high vacuum for *t* = 4 h at *T* = 250 °C prior to the measurement. To determine the specific surface *S_BET_*, the seven-point BET multi-point method in a relative pressure range of 0.02–0.25 was used.

Energy-dispersive X-ray fluorescence (XRF) analyses to determine the mass fractions of Bi and Ce were performed on a FISCHERSCOPE® X-RAY XAN® instrument and the evaluations were conducted with the program WinFTM® from Fischer Corp. The excitation voltage was *U* = 30 keV and the measuring time *t* = 60 s. The catalysts which were calcined at *T* = 800 °C were placed on top of a cellulose foil. Three repeated measurements at different points of the sample were employed and averaged in composition. Calibration of the measurement data was done by a standardless fundamental parameter method supplied by Fischer Corp (for more details see [22,23,24,25]).

## 3. Results

### 3.1. Raman and PXRD Analysis

**Figure 1:**
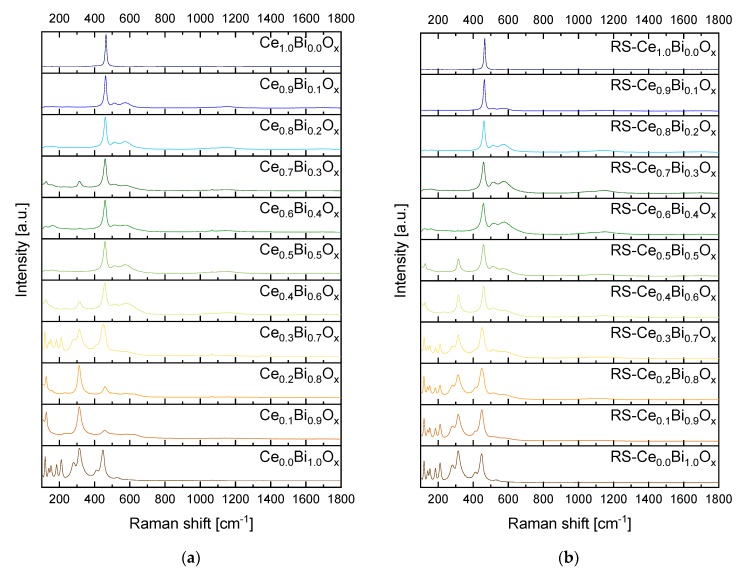
Raman spectra of the synthesized Bi-Ce-mixed oxides with varying Bi-Ce-molar ratios in 10 mol% steps. Each sample is measured three times. Shown are representative spectra of these measurements. All spectra are normalized for comparison. Oxides have been calcined at *T* = 800 °C. Laser wavelength is *λ* = 532 nm. (**a**) Normal synthesis route; (**b**) Reverse strike synthesis route.

As depicted in the Raman spectra in Figure 1, the synthesized Ce_1.0_Bi_0.0_O_x_ (pure ceria) samples for both co-precipitation routes show one band at ν = 462 cm^−1^. Note that with “x” we denote in the following that the amount of oxygen is variable and depending on preparation conditions, whereas the other subscripts in the all formulae represent the molar composition. The band at ν = 462 cm^−1^ was attributed to the F_2g_ mode of CeO_2_ [26]. This confirms that the pure CeO_2_ was synthesized in both cases without any defect structure. For the Ce_0.0_Bi_1.0_O_x_ (pure bismuth(III) oxide) all bands typical for α-Bi_2_O_3_ were observed for both synthesis methods. These bands were the following one: ν = 120 cm^−1^, 140 cm^−1^, 152 cm^−1^, 184 cm^−1^, 212 cm^−1^, 281 cm^−1^, 314 cm^−1^, 411 cm^−1^, 449 cm^−1^, and 532 cm^−1^ [26,27,28,29]. For both synthesis routes, with increasing Ce content the F_2g_ band of CeO_2_ dominates the Raman spectra but with a higher F_2g_ band FWHM (full width at half maximum, see supplementary information Table S1) compared to the intense F_2g_ band of the Ce_1.0_Bi_0.0_O_x_ spectra.

Figure 1a shows the Raman spectra of the normal co-precipitation route. In the range from Ce_0.1_Bi_0.9_O_x_ to Ce_0.4_Bi_0.6_O_x_ bands for β- and α-Bi_2_O_3_ can be identified. Especially, the spectra of the compositions Ce_0.1_Bi_0.9_O_x_, Ce_0.2_Bi_0.8_O_x_, and Ce_0.4_Bi_0.6_O_x_ show the band profile of β-Bi_2_O_3_ with the following typical bands: ν = 125 cm^−1^, 232 cm^−1^, 314 cm^−1^, and 462 cm^−1^ [29]. Through position-resolved Raman micro-spectroscopy at different locations of the samples, we found that the samples were inhomogeneous with regards to phase composition. Areas with predominantly α-Bi_2_O_3_ and areas with β-Bi_2_O_3_ mixed with CeO_2_ were observed. The F_2g_ band of CeO_2_ at ν = 464 cm^−1^ and the band of β-Bi_2_O_3_ at ν = 466 cm^−1^ are overlapping [26,29]. Therefore, it is hardly possible to distinguish between CeO_2_ and β-Bi_2_O_3_ in the range of high Bi content. With increasing Ce content, the typical bands for the bismuth oxides are disappearing and the F_2g_ band of CeO_2_ dominates the spectra except the spectrum for Ce_0.7_Bi_0.3_O_x_. This spectrum contains bands for β-Bi_2_O_3_ and CeO_2_. The spectra for Ce_0.8_Bi_0.2_O_x_ and Ce_0.9_Bi_0.1_O_x_ show two broad bands at ν = 510 cm^−1^ and 570 cm^−1^. According to Schilling et al. these bands can be assigned to oxygen vacancy formation $Ce^{3+}O_{7}Vö$ and $Ce^{4+}O_{7}Vö$ [26]. Figure 1b shows the Raman spectra of the reverse strike co-precipitation samples. The oxides RS-Ce_0.1_Bi_0.9_O_x_, RS-Ce_0.2_Bi_0.8_O_x_, and RS-Ce_0.3_Bi_0.7_O_x_ show clearly the typical bands originating from α-Bi_2_O_3_. The two oxides RS-Ce_0.4_Bi_0.6_O_x_ and RS-Ce_0.5_Bi_0.5_O_x_ have the typical F_2g_ band of CeO_2_ and the bands of β-Bi_2_O_3_. From RS-Ce_0.6_Bi_0.4_O_x_ to RS-Ce_0.9_Bi_0.1_O_x_ only the F_2g_ band and the two bands at ν = 510 cm^−1^ and 570 cm^−1^ resulting from the defects of the fluorite-type structure are to be recognized.

**Figure 2:**
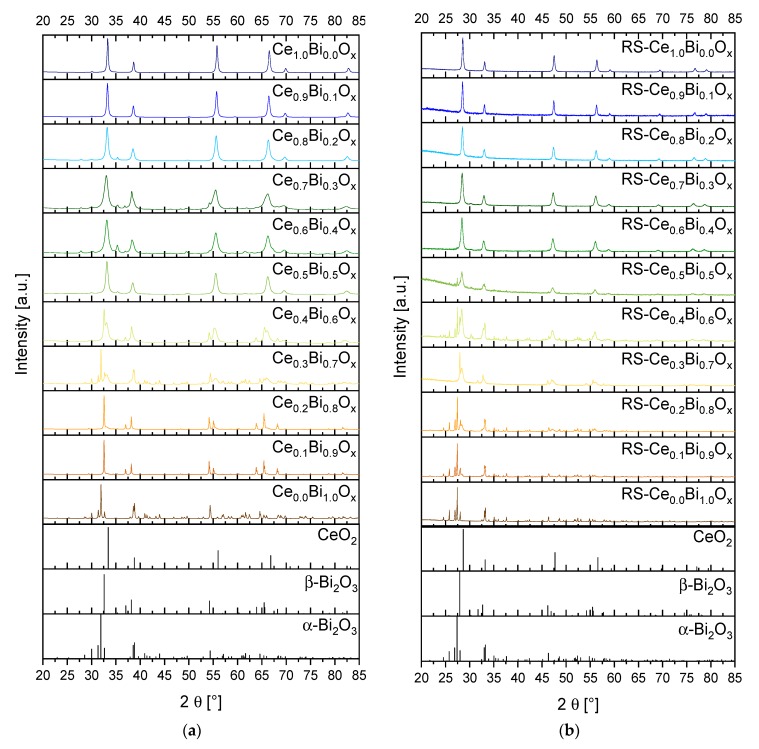
Powder X-ray diffraction (PXRD) pattern of the synthesized Bi-Ce mixed oxides with varying Bi-Ce molar ratios in 10 mol% steps. Oxides calcined at *T* = 800 °C. (**a**) Normal synthesis route. Measured with Co-K_α_ radiation, *λ* = 178.92 nm; (**b**) Reverse strike synthesis route. Measured with Cu-K_α_ radiation, *λ* = 154.06 nm. Reference files for CeO_2_: ICDD#75-76; α-Bi_2_O_3_: ICDD#71-2274; β-Bi_2_O_3_: ICDD#78-1793.

**Figure 3:**
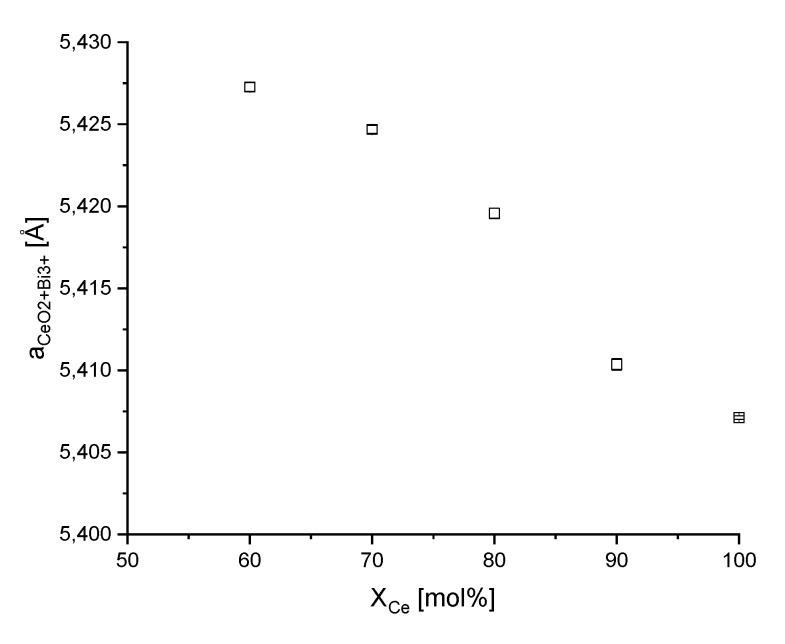
Lattice parameter *a* with standard deviation (STD) of *a* of cubic fluorite-type Bi^3+^-doped CeO_2_ phase in dependence of the nominal molar Ce content determined by Rietveld refinement with the program TOPAS [30].

Figure 2 presents the PXRD pattern of the synthesized samples for both synthesis routes. In general, the PXRD data confirm the results from Raman analyses. For both synthesis routes, the PXRD pattern shows that the oxides Ce_0.0_Bi_1.0_O_x_ adopt the monoclinic α-Bi_2_O_3_ crystal structure with space group P2_1_/c. The synthesized Ce_1.0_Bi_0.0_O_x_ crystallises cubic in a fluorite-type structure with the space group $\mathrm{Fm}\bar{3}m$. The PXRD pattern of the mixed oxides with increasing Bi content shows that, additionally to the reflections of ceria lattice structure, diffraction signals of the monoclinic α-Bi_2_O_3_ structure and of the tetragonal β-Bi_2_O_3_ with space group P$\bar{4}$2_1_c are appearing. For the normal synthesis route, only for the oxides Ce_0.8_Bi_0.2_O_x_ and Ce_0.9_Bi_0.1_O_x_ a single phase of Bi^3+^-doped CeO_2_ was observed. The lattice parameter *a* and the mass fraction of Bi^3+^ in the CeO_2_ lattice have been determined by Rietveld refinement. Doping CeO_2_ with Bi^3+^ increases the lattice parameter *a* from 5.4110(1) Å for CeO_2_ to 5.4174(1) Å for Ce_0.9_Bi_0.1_O_x_ and further on 5.4235(1) Å for Ce_0.8_Bi_0.2_O_x_. In contrast, with the reverse strike synthesis route in the range of pure ceria to RS-Ce_0.6_Bi_0.4_O_x_ a single Bi^3+^-doped CeO_2_ crystalline phase was observed, and the range of existence was thus broader than for the normal precipitation process. Figure 3 shows the lattice parameter *a* from Rietveld refinement of the Bi^3+^-doped CeO_2_ phases, and in Tables S2 and S3 all refined parameters are specified in detail. The insertion of Bi^3+^ ions into the CeO_2_ lattice increased the lattice parameter. For RS-Ce_0.6_Bi_0.4_O_x_ the lattice parameter *a* was 5.4273(3) Å. The lattice parameter *a* for the synthesized pure RS-CeO_2_ was 5.4071(1) Å.

### 3.2. XRF and BET Results

To verify the nominal Bi and Ce metal mass percentages of the oxides, i.e. without inclusion of oxygen content, XRF measurements were performed. Table 2 shows the acquired mass percentages. Note that there were no larger deviations between nominal and the expected mass percentages of the samples. Furthermore, the mass percentages of Bi and Ce differed not more than 2% between the two synthesis routes.

Table 3 shows the total surface area *S_BET_* of the synthesized samples determined by N_2_ physisorption experiments. The *S_BET_* was determined by the multi-point BET method. For the normal precipitation route, the pure Bi_2_O_3_ had with *S_BET_* = 0.4 m^2^∙g^−1^ the lowest specific surface. With increasing Ce content, the *S_BET_* increases as well. The highest surface area was observed for the pure CeO_2_ with *S_BET_* = 26.6 m^2^·g^−1^. For the compositions Ce_0.6_Bi_0.4_O_x_ with *S_BET_* = 18.7 m^2^·g^−1^ and Ce_0.8_Bi_0.2_O_x_ with *S_BET_* = 13.1 m^2^·g^−1^ also relatively high surface areas within this test series were observed. Surprisingly, the composition Ce_0.7_Bi_0.3_O_x_ has only a *S_BET_* of 6.5 m^2^·g^−1^. The differences in the observed Raman and PXRD data of this composition compared to others also reflected this behavior.

For the reverse strike co-precipitation route, as Ce content increased the *S_BET_* also increased. The maximum of the surface area was reached at a Ce content of 80 mol% with *S_BET_* = 21.3 m^2^·g^−1^. A further increase of the Ce content led to a decrease in *S_BET_*. For pure CeO_2_ prepared by the reverse-strike precipitation method only *S_BET_* = 6.2 m^2^·g^−1^ was observed.

### 3.3. OSC_dyn_ and TGA Results

The dynamic oxygen storage capacities *OSC_dyn_* were measured via thermogravimetric analyses in defined gas atmospheres and are shown in Table 4. First, each sample was heated with a specific heating rate under nitrogen for desorption of compounds like water, and to reduce the sample to substoichiometric oxides. Second, the sample was cooled under synthetic air. At this period, the sample was reoxidized. These two steps were repeated. The second reoxidation step was used for calculating the *OSC_dyn_* as difference in masses under the two atmospheres at specific temperatures (see chapter 2.2. for details). The *OSC_dyn_* is an important factor for the Diesel soot oxidation because the total available surface lattice oxygen is related to the *OSC* [31]. For the normal synthesis route, Ce_0.8_Bi_0.2_O_x_ had the highest *OSC_dyn_* with 34.0 µmol_O2_∙g_Kat_^−1^ and Ce_0.0_Bi_1.0_O_x_ revealed the lowest *OSC_dyn_* with 1.6 µmol_O2_∙g_cat_^−1^. For this synthesis route, there was no obvious correlation between the composition of oxide and the determined *OSC_dyn_*. In contrast to that, the *OSC_dyn_* for the reverse strike synthesis route was lower. Nevertheless, a correlation between the composition of oxide and the determined *OSC_dyn_* seems to exist. Starting from pure bismuth oxide the *OSC_dyn_* increased with an increase in Ce content. The composition RS-Ce_0.8_Bi_0.2_O_x_ with a value of *OSC_dyn_* = 26.4 µmol_O2_∙g_cat_^−1^ seems to represent the maximum in this synthesis series. After that, the *OSC_dyn_* decreased to a value of 1.9 µmol_O2_∙g_cat_^−1^ for RS-Ce_1.0_Bi_0.0_O_x_. Furthermore, it is to be noted that the *OSC_dyn_* in the intermediate range between the pure cerium oxides and bismuth oxides were different for both synthesis routes. The *OSC_dyn_* for the reverse strike co-precipitation of pure bismuth oxide was eight times higher than for the normal synthesis route and the reverse strike co-precipitation of pure cerium oxide was 13 times smaller than the normal synthesis route. The *S_BET_* reflected the same trend. There, the *S_BET_* for the reverse strike route was five times higher than the normal synthesis route. This shows clearly that the *OSC_dyn_* really represents a surface parameter, in this case the surface oxygen lattice ions to be utilized for oxidation processes.

In thermogravimetric (TGA) measurement data, the *T_50_* value is used to characterise the activity for the Diesel soot oxidation. The *T_50_* value describes the temperature where 50% of the model soot is oxidized in a model gas mixture of defined flow rate and heating rate. This value is commonly used in the literature. Table 5 presents the determined *T_50_* values. Only the *T_50_* values of tight soot-catalyst contact conditions were presented, but also other contact modes were prepared and screened.

The lowest *T_50_* value was reached for the compositions Ce_0.8_Bi_0.2_O_x_ and RS-Ce_0.8_Bi_0.2_O_x_. Like for the *S_BET_* and *OSC_dyn_*, there was a less distinct correlation between *T_50_* and the composition of the oxide for the normal synthesis route indicating nuisance factors in the syntheses. In contrast to the normal synthesis route, there was a clear dependency in *T_50_* values as a function of oxide composition for the reverse strike synthesis route. Starting from pure bismuth oxide the *T_50_* decreased with higher Ce content to a minimum for composition RS-Ce_0.8_Bi_0.2_O_x_ with a *T_50_* value of 444 °C. After that, the *T_50_* value increased again to 586 °C for RS-Ce_1.0_Bi_0.0_O_x_. In general, and for both synthesis routes, the *T_50_* values of pure CeO_2_ and Bi_2_O_3_ were higher than that of the mixed oxides.

## 4. Discussion

### 4.1. Normal Synthesis Route

**Figure 4:**
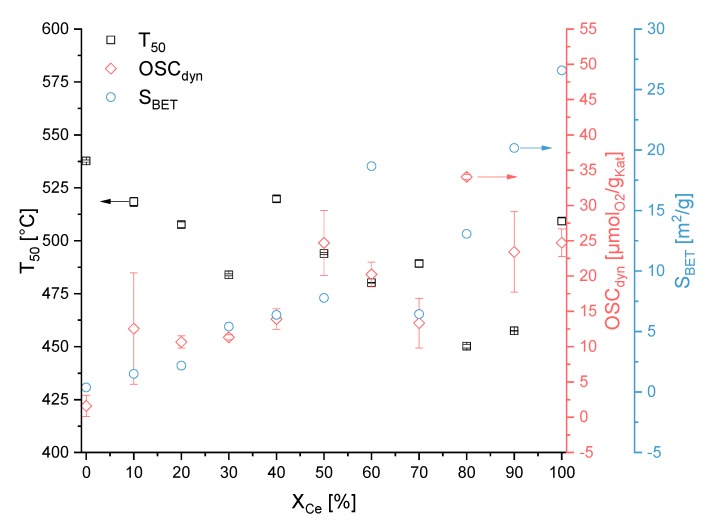
*T_50_* (tight contact), *S_BET_* and *OSC_dyn_* values in dependence of the nominal molar Ce content for samples prepared by normal precipitation. Shown are duplicate measurements together with their standard deviation.

PXRD data refinements reveal that in compositions with high Bi content two crystalline Bi_2_O_3_ phases can be detected, i.e., monoclinic α-Bi_2_O_3_ and tetragonal β-Bi_2_O_3_. Additionally reflections representing about 5m% bismutite Bi_2_(CO_3_)O_2_ can also be detected in PXRD determined by Rietveld refinement. Confirmation of these results is achieved by Raman spectroscopy. With increasing Ce content up to the composition Ce_0.7_Bi_0.3_O_x_, a β-Bi_2_O_3_ phase is present. One reason for this observation could be the initial stages of precipitation of the oxides. From a visual inspection of the precipitation process, we find the beginning of precipitation for bismuth oxide at Ph < 1 and for cerium oxide near to pH = 4. After calcination, the α-Bi_2_O_3_ polymorph should be formed as this is the thermodynamic stable form [32]. Additionally, it is known that hydrated bismuth oxides oligomerise in aqueous acidic media [18]. In our case, also the β-Bi_2_O_3_ form is present. The oligomerisation of the hydrated bismuth oxides at low pH values and the incorporation of cerium oxide into the crystal lattice, may both serve as an explanation for the observation of the tetragonal β-Bi_2_O_3_ form. Another reason is that the formation of β-Bi_2_O_3_ may result from decomposition of bismutite [33]. With increasing Ce content, the F_2g_ mode in the Raman spectra gets the dominant band. Above a Ce content of 50 mol% there are two more bands to be assigned to CeO_2_. The band at ν ~ 510 cm^−1^ is caused by the formation of Ce^3+^ ions in combination with oxygen vacancies. The band at ν ~ 570 cm^−1^ originates from oxygen vacancies in combination with Ce^4+^ ions (see above and ref. [19]). From Figure 1 it can be seen that these two defect bands are most noticeable for the compositions Ce_0.8_Bi_0.2_O_x_ and Ce_0.9_Bi_0.1_O_x_ and that in the samples with these compositions only the Bi^3+^-doped CeO_2_ phase is verifiable. Interestingly, these two compositions have, for this synthesis route, the lowest *T_50_* values and highest *OSC_dyn_* values compared to the other compositions. This is illustrated in Figure 4.

### 4.2. Reverse Strike Synthesis Route

**Figure 5:**
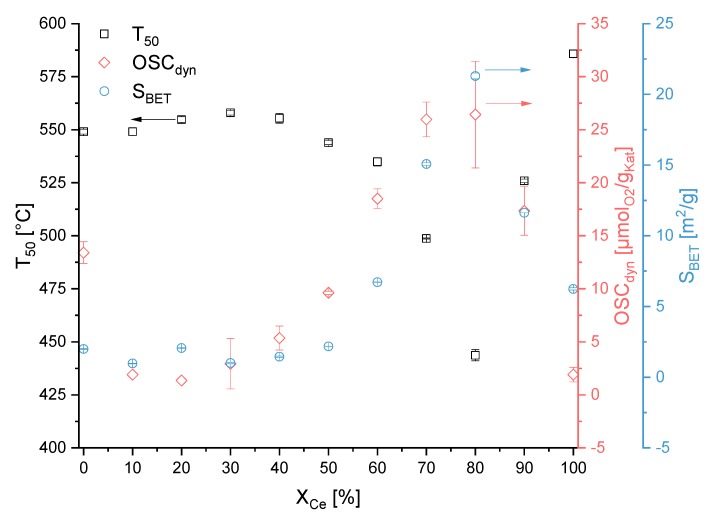
*T_50_* (tight contact), *S_BET_* and *OSC_dyn_* values in dependence of the nominal molar Ce content for samples prepared by reverse strike precipitation. Shown are duplicate measurements with their standard deviation.

To avoid the oligomerisation of the hydrated bismuth oxides in aqueous acidic media and the sequential precipitation of oxides with different compositions, i.e., cerium and bismuth oxides separately, the reverse strike co-precipitation method is applied. During this synthesis route, the premixed acidic precursor solutions are added to the basic precipitation agent solution. The precipitation is stopped at pH = 6. Therefore, an acidic pH range is avoided. According to the PXRD data, see Figure 2b, below a molar fraction of 60 mol% Ce only a Bi^3+^-doped CeO_2_ phase is observed. These results are in accordance to the results of *Hund et al*. [15]. Compared to the normal synthesis route, with the reverse strike route the mixed oxide phase extends to even lower Ce fractional amounts. These results are supported by Raman analyses (see Figure 1b). Additionally, only bismutite is present in the Raman spectra of Ce_0.2_Bi_0.8_O_x_ to Ce_0.8_Bi_0.2_O_x_ as an additional phase in a minor amount. Furthermore, the two defect bands at ν = 510 cm^−1^ and ν = 570 cm^−1^ are very prominent within the composition range of Ce_0.6_Bi_0.4_O_x_ to Ce_0.8_Bi_0.2_O_x_. At higher Ce content the intensity of the two defect bands decreases again, and are not to be detected in the Raman spectrum of Ce_1.0_Bi_0.0_O_x_. In Section 3.1. we concluded that the formation of oxygen sublattice defects in ceria of fluorite-structure type may influence the activity of the catalyst for the Diesel soot oxidation. The more pronounced the defect structure is, the higher the activity will be for Diesel soot oxidation. In Figure 5, the correlation between *T_50_*, *OSC_dyn_* and *S_BET_* is presented for the samples of this synthesis route. For this synthesis series, a clear correlation between activity and characterization data can be seen. Starting from pure ceria with increasing Bi content, *T_50_* values decrease revealing that the soot oxidation activity increases. At the same time, both *OSC_dyn_* and *S_BET_* increase. This trend reaches its maximum – low *T_50_* value and high *OSC_dyn_* and *S_BET_* – at 80 mol% Ce content. This is in accordance with the described effect of the Raman spectra with the two defect bands at ν = 510 cm^−1^ and ν = 570 cm^−1^. At still lower Ce content, the *T_50_* values increase, and the *OSC_dyn_* and *S_BET_* values decrease again. In the same sequence, the intensities of the two defect bands in Raman spectra decrease. The two defect bands are not detected in the Raman spectrum of pure ceria.

## 5. Conclusions

In this paper, two automated routes of co-precipitation methods to synthesize a complete series of Bi-Ce mixed oxides Ce_1−x_Bi_x_O_2−x/2_ (0 ≤ x ≤ 1) for catalytic oxidation of Diesel soot are described: The normal synthesis and the reverse strike synthesis. The synthesized oxides are characterized by PXRD, Raman spectroscopy, and nitrogen adsorption to measure the specific surface. Furthermore, the dynamic oxygen storage capacities *OSC_dyn_* are determined by TGA methods. The catalytic activities are also measured by TGA under continuous gas flow conditions and specified by *T_50_* values. Raman spectroscopy reveals that, with an increasing Ce content, an oxygen defect structure of the fluorite-type ceria lattice as a result of Bi^3+^ insertion into the cation sublattice occurs (in Kröger-Vink notation, see equation 1):

(1) $$Bi_{2}O_{3}\left(CeO_{2}\right)⇌2Bi_{Ce}^{\prime }+Vö+3O_{O}^{x}$$

At least two bands resulting from oxygen defects of the ceria lattice are observed in the Raman spectra. Additionally, a relation between *OSC_dyn_*, *S_BET_* and *T_50_* values is found indicating that catalytic activity for Diesel soot oxidation and defect structure are highly correlated. This relation is very striking for the mixed oxides, which are synthesized through the reverse strike method, but less distinct for samples prepared by the normal precipitation route. Here, the maximal catalytic activity with the maximal *OSC_dyn_* and *S_BET_* is reached at the maximal intensity of the two defect bands in the Raman spectra at the optimal composition of Ce_0.8_Bi_0.2_O_1.9_. Compared to the pure reverse strike co-precipitated CeO_2_ RS-Ce_1.0_Bi_0.0_O_x_, the *T_50_* decreases from 586 °C to 444 °C. This is an improvement of Δ*T_50_* ≈ 140 °C. Simultaneously, the *OSC_dyn_* increases from 1.9 µmol_O2_∙g_cat_^−1^ for the RS-Ce_1.0_Bi_0.0_O_x_ to 26.4 µmol_O2_∙g_cat_^−1^ for the RS-Ce_0.8_Bi_0.2_O_x_. This is an increase of Δ*OSC_dyn_* = 24.5 µmol_O2_∙g_cat_^−1^. Additionally, the *S_BET_* also increases from 6.2 m^2^∙g^−1^ for the RS-Ce_1.0_Bi_0.0_O_x_ to 21.3 m^2^∙g^−1^ for the RS-Ce_0.8_Bi_0.2_O_x_. This is an increase of Δ*S_BET_* = 15,1 m^2^∙g^−1^. PXRD data reveal that with this composition we are still in the phase field of a cubic defect structure of the fluorite-type structure and that the field of stability for this mixed oxide phase is limited to the compositional range 0 ≤ x ≤ 0.4. At higher x also pure bismuth oxide phases are formed. Due to ionic radii differences (r(Bi^3+^) = 1.03 Å, r(Ce^3+^) = 1.01 Å, r(Ce^4+^) = 0.87 Å, all for CN = 6 [34]) the incorporation of Bi^3+^ into the ceria lattice Ce^4+^(O^2−^)_2_ “widens” the crystal lattice as can be seen from the increase in the lattice parameter *a*. Additionally, due to the lone pair configuration of Bi^3+^, a local distortion of the cation coordination environment by oxygen anions occurs. Both factors facilitate the incorporation of larger Ce^3+^ ions into the cationic Ce^4+^ sublattice, thus, increasing the oxygen storage capacity, as for each O^2−^ removed for charge neutrality two Ce^4+^ ions have to be reduced to Ce^3+^ (so-called Schottky defect set in square brackets, see equation 2):

(2) $$2CeO_{2}\left(CeO_{2}\right)⇌\left[2Ce_{Ce}^{\prime }+Vö\right]+3O_{O}^{x}+\frac{1}{2}O_{2}$$

As on the other side, too much Bi^3+^ is destabilising the lattice, as seen from the crystallisation of additional bismuth oxide phases. There is an optimum of oxygen storage capacity and thus minimum of *T_50_* value, in this case at x = 0.2.

## Figures and Tables

**Table 1:** Dispensing and aspirating speeds of the pipetting steps. The 4-needle head (4NH) of the synthesis robot Chemspeed Accelerator SLT106 is used for liquid dosing.

Solution	Dispensing Speed [mL·min^−1^]	Aspirating Speed [mL·min^−1^]
0.5 M Ce(NO_3_)_3_·6H_2_O	40	40
0.5 M Bi(NO_3_)_3_·5H_2_O	40	40
premixed nitrate solution	40	40
1 M (NH_4_)_2_CO_3_	40	80

**Table 2:** Mass percentages m% of the synthesized mixed oxides determined by X-ray fluorescence (XRF) analysis. Shown are the averaged values of three repeated measurements of one sample at different areas with standard deviations.

Composition of Oxide	Nominal Mass Percentages		Measured Mass Percentage			
Formulae			Normal Synthesis		Reverse Strike Synthesis	
	m%_Ce_	m%_Bi_	m%_Ce_	m%_Bi_	m%_Ce_	m%_Bi_
Ce_0.0_Bi_1.0_O_x_	0.00	100.00	0.00 ± 0.00	100.0 ± 0.00	0,00 ± 0.00	100.0 ± 0.00
Ce_0.1_Bi_0.9_O_x_	7.59	92.41	6.89 ± 0.17	92.85 ± 0.15	7.52 ± 0.15	92.48 ± 0.15
Ce_0.2_Bi_0.8_O_x_	15.59	84.41	14.36 ± 0.16	85.26 ± 0.16	15.15 ± 0.44	84.85 ± 0.44
Ce_0.3_Bi_0.7_O_x_	24.05	75.95	22.86 ± 0.26	76.51 ± 0.30	23.52 ± 0.04	76.48 ± 0.04
Ce_0.4_Bi_0.6_O_x_	33.00	67.00	31.71 ± 0.21	67.64 ± 0.26	30.07 ± 0.55	69.31 ± 0.45
Ce_0.5_Bi_0.5_O_x_	42.49	57.51	45.52 ± 1.33	54.18 ± 1.64	44.09 ± 0.19	55.91 ± 0.19
Ce_0.6_Bi_0.4_O_x_	52.56	47.44	57.13 ± 0.44	42.27 ± 0.19	55.58 ± 0.20	44.42 ± 0.20
Ce_0.7_Bi_0.3_O_x_	63.29	36.71	60.64 ± 0.74	38.50 ± 0.79	58.25 ± 0.37	40.71 ± 0.36
Ce_0.8_Bi_0.2_O_x_	74.72	25.28	75.42 ± 0.75	23.85 ± 0.27	78.66 ± 0.10	21.34 ± 0.10
Ce_0.9_Bi_0.1_O_x_	86.93	13.07	85.64 ± 0.40	13.96 ± 0.16	89.17 ± 0.09	10.83 ± 0.09
Ce_1.0_Bi_0.0_O_x_	100.00	0.00	100.0±0.00	0.00 ± 0.00	100.0 ± 0.00	0.00 ± 0.00

**Table 3:** Calculated specific surface areas according to Brunauer, Emmett, and Teller (*S_BET_)* of the synthesized mixed Bi-Ce oxides determined by nitrogen adsorption measurements. Oxides are calcined at *T* = 800 °C.

Composition of Oxide	S_BET,normal synthesis_ [m^2^·g^−1^]	S_BET,reverse strike synthesis_ [m^2^·g^−1^]
Ce_0.0_Bi_1.0_O_x_	0.4	2.0
Ce_0.1_Bi_0.9_O_x_	1.5	1.0
Ce_0.2_Bi_0.8_O_x_	2.2	2.1
Ce_0.3_Bi_0.7_O_x_	5.4	1.0
Ce_0.4_Bi_0.6_O_x_	6.4	1.5
Ce_0.5_Bi_0.5_O_x_	7.8	2.2
Ce_0.6_Bi_0.4_O_x_	18.7	6.7
Ce_0.7_Bi_0.3_O_x_	6.5	15.1
Ce_0.8_Bi_0.2_O_x_	13.1	21.3
Ce_0.9_Bi_0.1_O_x_	20.2	11.6
Ce_1.0_Bi_0.0_O_x_	26.6	6.2

**Table 4:** Calculated dynamic oxygen storage capacity *(OSC_dy_n)* of the synthesized mixed oxides determined by thermogravimetric analysis (TGA). Measurement temperature in the range of *T* = 150–700 °C with heating rate of *r* = 5 °C∙min^−1^ in synthetic air and nitrogen. The average of double determination with the standard deviation is shown.

Composition of Oxide	OSC_dyn,normal synthesis_ [µmol_O2_·g_cat_^−1^]	OSC_dyn,reverse strike synthesis_ [µmol_O2_·g_cat_^−1^]
Ce_0.0_Bi_1.0_O_x_	1.6 ± 1.5	13.4 ± 1.0
Ce_0.1_Bi_0.9_O_x_	12.6 ± 7.9	1.9 ± 0.0
Ce_0.2_Bi_0.8_O_x_	10.7 ± 0.9	1.4 ± 0.0
Ce_0.3_Bi_0.7_O_x_	11.3 ± 0.3	2.9 ± 2.4
Ce_0.4_Bi_0.6_O_x_	13.9 ± 1.5	5.4 ± 1.1
Ce_0.5_Bi_0.5_O_x_	24.7 ± 4.6	9.6 ± 0.2
Ce_0.6_Bi_0.4_O_x_	20.3 ± 1.8	18.5 ± 0.9
Ce_0.7_Bi_0.3_O_x_	13.3 ± 3.5	26.0 ± 1.6
Ce_0.8_Bi_0.2_O_x_	34.0 ± 0.4	26.4 ± 5.0
Ce_0.9_Bi_0.1_O_x_	23.4 ± 5.7	17.3 ± 2.3
Ce_1.0_Bi_0.0_O_x_	24.7 ± 2.0	1.9 ± 0.7

**Table 5:** *T_50_* values for Diesel soot oxidation in tight contact mode of the synthesized mixed oxides determined by TGA. Measurement temperature in the range of *T* = 25–700 °C with heating rate of *r* = 5 °C∙min^−1^ in synthetic air. The averages of double determination with standard deviations are given.

Composition of Oxide	T_50.normal synthesis_ [°C]	T_50.reverse strike synthesis_ [°C]
Ce_0.0_Bi_1.0_O_x_	537.7 ± 0.5	549.1 ± 1.1
Ce_0.1_Bi_0.9_O_x_	518.4 ± 2.1	549.1 ± 1.8
Ce_0.2_Bi_0.8_O_x_	507.6 ± 1.4	554.8 ± 1.3
Ce_0.3_Bi_0.7_O_x_	483.9 ± 0.5	558.0 ± 1.0
Ce_0.4_Bi_0.6_O_x_	519.8 ± 1.4	555.4 ± 2.3
Ce_0.5_Bi_0.5_O_x_	494.0 ± 0.5	543.9 ± 1.0
Ce_0.6_Bi_0.4_O_x_	480.2 ± 0.2	534.9 ± 2.0
Ce_0.7_Bi_0.3_O_x_	489.3 ± 1.5	498.7 ± 0.2
Ce_0.8_Bi_0.2_O_x_	450.3 ± 0.8	443.7 ± 2.7
Ce_0.9_Bi_0.1_O_x_	457.5 ± 0.1	525.9 ± 0.8
Ce_1.0_Bi_0.0_O_x_	509.2 ± 1.6	585.8 ± 1.7

## Supplementary Materials

The following are available online at https://www.mdpi.com/1996-1944/13/6/1369/s1. Table S1: FWHM (full width at half maximum) of the F_2g_ band from the CeO_2_ lattice of Raman spectra. Given is the average of three repeated measurements and the standard deviation. FWHM is determined with the software Origin Pro 2019b; Table S2: Results of Rietveld-Refinement of the normal precipitation route. Samples are calcined by T = 800 °C; Table S3: Results of Rietveld-Refinement of the reverse strike precipitation route. Samples are calcined by T = 800 °C.
