# Supplementary material for: Synthesis and Characterization of Bismuth-Cerium Oxides for the Catalytic Oxidation of Diesel Soot
Source: Materials (Basel). 2020 Mar 18;13(6):1369. doi: 10.3390/ma13061369 (PMC7143761; doi:10.3390/ma13061369)
Supplement: Supplementary file 1 [file materials-13-01369-s001.pdf]

materials

MDPI

Article

Synthesis and Characterization of Bismuth-Cerium Oxides for the Catalytic Oxidation of Diesel Soot

Sabrina C. Hebert and Klaus Stöwe *

Faculty of Natural Science, Professorship of Chemical Technology, Chemnitz University of Technology, Chemnitz, 09111, Germany; sabrina-christina.hebert@chemie.tu-chemnitz.de
* Correspondence: klaus.stöwe@chemie.tu-chemnitz.de

FWHM of CeO_2-F_{2g} band (full width at half maximum) of Raman spectra:

Table 1. FWHM (full width at half maximum) of the F_{2g} band from the CeO_2 lattice of Raman spectra. Given is the average of three repeated measurements and the standard deviation. FWHM is determined with the software Origin Pro 2019b.

Composition of Oxide/Formulae	$FWHM_{Normal\ synthesis}$ [cm^{-1}]	$FWHM_{Reverse\ strikel\ synthesis}$ [cm^{-1}]
$Ce_{0.0}Bi_{1.0}O_x$	/	/
$Ce_{0.1}Bi_{0.9}O_x$	/	/
$Ce_{0.2}Bi_{0.8}O_x$	/	/
$Ce_{0.3}Bi_{0.7}O_x$	/	/
$Ce_{0.4}Bi_{0.6}O_x$	26(2)	24(3)
$Ce_{0.5}Bi_{0.5}O_x$	26(3)	21.4(2)
$Ce_{0.6}Bi_{0.4}O_x$	24(2)	20.7(3)
$Ce_{0.7}Bi_{0.3}O_x$	23(2)	21(1)
$Ce_{0.8}Bi_{0.2}O_x$	16(1)	18.1(6)
$Ce_{0.9}Bi_{0.1}O_x$	12.1(5)	14(1)
$Ce_{1.0}Bi_{0.0}O_x$	9.8(1)	10.1(3)

Table 2. Results of Rietveld-Refinement of the normal precipitation route. Samples are calcined by T = 800 °C. .

**Composition oxide/formulae**	$\mathbf{Ce_{0.0}Bi_{1.0}O_x}$	$\mathbf{Ce_{0.1}Bi_{0.9}O_x}$	$\mathbf{Ce_{0.2}Bi_{0.8}O_x}$	$\mathbf{Ce_{0.3}Bi_{0.7}O_x}$	$\mathbf{Ce_{0.4}Bi_{0.6}O_x}$	$\mathbf{Ce_{0.5}Bi_{0.5}O_x}$	$\mathbf{Ce_{0.6}Bi_{0.4}O_x}$	$\mathbf{Ce_{0.7}Bi_{0.3}O_x}$	$\mathbf{Ce_{0.8}Bi_{0.2}O_x}$	$\mathbf{Ce_{0.9}Bi_{0.1}O_x}$	$\mathbf{Ce_{1.0}Bi_{0.0}O_x}$
CeO_2 + Bi [%]		28(1)	33(2)	22(2)	75(2)	51(2)	52(2)	80(1)	96(1)	100(1)	
a [Å]		5.441(2)	5.442(5)	5.448(6)	5.440(2)	5.436(2)	5.434(3)	5,431(2)	5.423(1)	5.4174(6)	
w_{Ce} [%]		66(4)	79.2(4)	86(5)	67(2)	63.3(2)	79.8(2)	68.9(2)	78.0(9)	89.9(7)	
w_{Bi} [%]		34(4)	20.8(4)	14(5)	33(2)	36.7(2)	20.2(2)	31.1(2)	22.0(9)	10.1(7)	
Cry Size L [nm]		16.4(2)	18.3(5)	20.1(7)	23.2(5)	26.2(4)	19.1(2)	29.8(7)	34.7(5)	39.1(2)	
Bismutite [%]	1.5(1)	0.5(1)	4.1(2)	4.2(2)		2.7(1)	9.0(1)	5.1(2)	4.2(1)		
a [Å]	5.487(5)	5.487(4)	5.450(1)	5.45(1)		5.47(3)	5.463(6)	5.44(2)	5.46(1)		
b [Å]	27.1(4)	27.4(2)	27.5(1)	27.5(1)		27.4(1)	27.36(3)	27.4(2)	27.36(9)		
c [Å]	5.58(2)	5.47(5)	5.45(1)	5.45(2)		5.45(5)	5.465(9)	5.44(2)	5.46(2)		
Cry Size L [nm]	56(9)	34(5)	17.4(9)	18.7(9)		33(2)	34.3(7)	10.2(5)	25(1)		
β-Bi_2O_3 [%]	2.5(2)	72(1)	8.4(2)	7.5(3)	24.7(3)		4.1(1)	14.7(2)			
a [Å]	7.7441(8)	7.7407(4)	7.739(6)	7.740(6)	7.731(2)		7.730(3)	7.728(3)			
c [Å]	5.616(2)	5.6411(5)	5.637(7)	5.636(7)	5.648(2)		5.633(9)	5.640(3)			
Cry Size L [nm]	94(10)	197(3)	40(1)	40(1)	72(2)		169(13)	68(2)			
α-Bi_2O_3 [%]	96(1)		42(1)	41(1)							
a [Å]	5.8504(5)		5.8562(9)	5.8562(9)							
b [Å]	8.1666(6)		8.161(1)	8.160(1)							
c [Å]	7.5111(8)		7.508(2)	7.508(1)							
β [°]	112.972(7)		112.98(2)	112.98(2)							
Cry Size L [nm]	213(3)		160(3)	160(3)							
CeO_2 [%]			12(1)	25(2)		46(2)	35(1)				100(1)
a [Å]			5.41(1)	5.422(7)		5.415(4)	5.409(5)				5.4109(5)
Cry Size L [nm]			17.2(6)	17.2(4)		22.2(3)	18.1(3)				46.5(2)
R_{WP}	10.014	7.826	6.600	6.562	6.009	4.939	4.940	5.680	5.382	5.933	5.068

Table 3. Results of Rietveld-Refinement of the reverse strike precipitation route. Samples are calcined by T = 800 °C.

Composition Oxide/Formulae	$Ce_{0.0}Bi_{1.0}O_x$	$Ce_{0.1}Bi_{0.9}O_x$	$Ce_{0.2}Bi_{0.8}O_x$	$Ce_{0.3}Bi_{0.7}O_x$	$Ce_{0.4}Bi_{0.6}O_x$	$Ce_{0.5}Bi_{0.5}O_x$	$Ce_{0.6}Bi_{0.4}O_x$	$Ce_{0.7}Bi_{0.3}O_x$	$Ce_{0.8}Bi_{0.2}O_x$	$Ce_{0.9}Bi_{0.1}O_x$	$Ce_{1.0}Bi_{0.0}O_x$
CeO_2 + Bi [%]				76(2)	33(1)		100(1)	100(1)	100(2)	100(3)	
a [Å]				5.442(3)	5.439(6)		5.427(3)	5.423(2)	5.420(3)	5.410(4)	
w_{Ce} [%]				37(4)	60(4)		52.3(2)	61.4(2)	74(2)	82(3)	
w_{Bi} [%]				63(4)	40(4)		47.7(2)	38.6(2)	26(2)	18(3)	
Cry Size L [nm]				18.0(2)	26.3(5)		26.6(2)	28.7(3)	38.9(7)	53(1)	
Bismutit [%]					6.1(2)						
a [Å]					5.28(2)						
b [Å]					27.44(8)						
c [Å]					5.66(2)						
Cry Size L [nm]					59(2)						
β-Bi_2O_3 [%]		14.6(3)	18.6(6)	24.2(3)	31(2)	94(4)					
a [Å]		7.7(2)	7.7(1)	7.731(4)	7.72(6)	7.68(3)					
c [Å]		5.5(2)	5.4(2)	5.644(3)	5.46(9)	5.45(4)					
Cry Size L [nm]		24.5(7)	25.0(8)	190(12)	24(1)	25.7(9)					
α-Bi_2O_3 [%]	100.0(5)	80.1(4)	61.5(3)		21.4(2)						
a [Å]	5.8456(9)	5.851(1)	5.851(1)		5.852(2)						
b [Å]	8.162(1)	8.155(2)	8.154(2)		8.155(3)						
c [Å]	7.506(1)	7.504(2)	7.504(2)		7.505(4)						
β [°]	112.98(1)	113.04(1)	113.01(2)		112.97(4)						
Cry Size L [nm]	9321(6728)	599(26)	546(29)		422(8)						
γ-Bi_2O_3 [%]		5.4(2)	4.1(3)		8.1(2)						
a [Å]		10.097(4)	10.10(1)		10.106(4)						
Cry Size L [nm]		555(138)	53(6)		224(3)						
CeO_2 [%]			16(1)			6(3)					100.0(4)
a [Å]			5.47(1)			5.41(2)					5.407(1)
Cry Size L [nm]			25.0(8)			120(4)					46.0(3)
RWP	6.839	8.205	7.574	3.990	5.366	4.001	6.329	5.089	4.678	7.916	4.425
